# Erenumab escalation in migraine - double dose without additional benefit - a retrospective experience

**DOI:** 10.1007/s13760-024-02603-z

**Published:** 2024-07-27

**Authors:** Simon Heintz, Peter Storch, Philipp Burow, Patricia Maier, Mark Obermann, Grit Stoessel, Torsten Kraya, Steffen Naegel

**Affiliations:** 1 Department of Neurology, University Hospital Halle, Martin Luther University Halle- Wittenberg, Wittenberg, Germany; 2 Headache Center Jena, Department of Neurology, University Hospital Jena, Jena, Germany; 3 Department of Neurology, Klinikum Weser-Egge, Höxter, Germany; 4 Department of Neurology, Hospital St. Georg, Leipzig, Germany; 5 Department of Neurology, Alfried Krupp Krankenhaus Essen, Alfried-Krupp-Straße 21, 45131 Essen, Germany

**Keywords:** Migraine, Erenumab, Real-world, Preventive medication

## Abstract

**Background:**

Erenumab is a monoclonal antibody specifically targeting the CGRP-receptor. Several studies showed efficacy and safety in patients with migraine. Less is known regarding dosage increase, especially in a difficult to treat patients. The aim of the study is to evaluate the increased dosage under real world conditions with particular focus on 70 mg non-responders.

**Methods:**

In a retrospective analysis, patients treated in tertiary headache centers (Halle or Jena, Germany) receiving 70 mg erenumab for at least 3 months with a dosage increase to 140 mg were analyzed. Data were evaluated regarding headache days, intake of acute medication, previous prophylaxis, and medication overuse. Baseline and all treatment intervals were determined as three-month periods.

**Results:**

Datasets of 52 migraine patients (90.4% women) aged between 22 and 78 years (mean 50.4 years, SD 12.1 years) were analyzed. At baseline (mean headache-days 15.67 ± 6.37) 51.9% met criteria for chronic migraine and 56% were currently overusing acute medication. While therapy with 70 mg showed significant improvement in headache days and 50% response, further improvement was not achieved for therapy escalation to 140 mg. The same applies to the secondary endpoints and covers the entire study population as well as the subgroups of chronic and episodic migraine. The 50% response of the 70 mg non-responders for escalation was only 5.14%.

**Conclusions:**

In this difficult-to-treat patient cohort we reconfirmed the effectiveness of erenumab, but could not detect any additional benefit for a dosage escalation from 70 mg to 140 mg erenumab.

## Background

Migraine is a primary headache disorder and is one of the leading neurological disorders causing disability worldwide [1]. Both non-medical and medical treatment strategies are necessary to improve quality of life in migraineurs. Besides effective acute therapy, prophylactic medication plays a crucial role. Several non-specific drugs for migraine prophylaxis such as beta- and calcium-channel blockers, anticonvulsants, and antidepressants are established [6]. Multiple studies have shown good efficacy and tolerability of antibodies against calcitonin gene-related peptide (CGRP) and the CGRP receptor [2]. Erenumab was the first CGRP based agent, approved 2018 by the European Medicines Agency, based on its efficacy for episodic and chronic migraine. Treatment with erenumab is safe, well tolerated and effective long-term in episodic and chronic migraine as proven in several randomized controlled studies [3–8].

There are two doses for erenumab (70 or 140 mg every 4 weeks). Initially, because only the 70 mg dosage was available, only patients with inadequate or waning effect were escalated to 140 mg. This invariably resulted in an increase in the duration of therapy and therapy-evaluation. However, current guidelines recommend a decision as to whether antibody therapy is sufficiently effective after a maximum of 3 months [9]. Very little is known about the efficacy of a dosage escalation from 70 to 140 mg, the timing of the right erenumab dosage in the right patient and maintenance of therapeutic effects over time.

We present real-world data collected at two Headache Centers in Germany recruiting difficult-to-treat migraine patients before and after dose escalation from 70 mg to 140 mg erenumab with follow-up of up to 6 months.

## Methods

In a retrospective chart analysis 52 outpatients receiving consecutive treatment with 70 and then 140 mg erenumab between 2018 and 2020 were identified. Treatment periods were predefined as follows: three months before initiation of 70 mg (pre70 = baseline (BL)), three months after 70 mg (post70). As not all patients were escalated exactly after three months, further defined periods were three months before and after dose escalation to 140 mg erenumab (pre140 and post140). Additionally, treatment after dose escalation was followed up for 6 months. Episodic and chronic migraine was diagnosed according to the International Classification of Headache Disorders 3rd edition (ICHD-3, [10]). Most patients previously failed (or had contraindications) ≥ 4 first-line migraine prophylactics (beta-blockers (propranolol or metoprolol), calcium channel blockers (flunarizine), tricyclic antidepressants (amitriptyline) and anticonvulsants (topiramate). Chronic migraine patients additionally failed treatment with onabotulinumtoxin A.

All patients included in the analysis were treated first with 70 mg erenumab and were then escalated to a 140 mg dosage in both study centers within the observation period. The dose increase followed the ineffectiveness of the 70 mg treatment, which was not predefined on the basis of formal criteria, but was rather subject to the physician’s judgment. Key prerequisite for the initiation of erenumab was the completion of a diagnostic headache diary covering at least 3 months before treatment initiation. Patients keeping up the headache diary for at least 3 additional months were included in the 140 mg erenumab group.

The following parameters were analyzed: headache days per month (primary endpoint: reduction of averaged three-month headache days in the defined time intervals), number of days of acute medication intake; 50% responder rates regarding the reduction of headache days; percentage of patients with medication overuse (MO) defined as ≥ 10 days of acute medication three months prior to erenumab initiation (secondary endpoints). We analyzed descriptively the number of previous treatment attempts with first line oral prophylactics as well as the number of discontinuation due to lack of tolerability, lack of effectiveness and contraindicated usability.

At each center, data were extracted from standardized documentation forms obtained during routine clinical outpatient visits by the local headache specialists (comprising data on patients’ history, medication regimen, previous treatments, medical and non-medical measures for migraine treatment). We analyzed patient headache diaries brought to those regular clinical visits. As patients did not all use the same headache diary, only data that were consistently documented were evaluated.

Data was scouted by different headache specialists and treating neurologists in Jena and Halle. A research associate used a designed data mask in both centers to enter the targeted data, later preparing it for statistical analysis. SPSS 25 (International Business Machines Corporation, Armonk, New York, USA) was used for analysis. The Greenhouse-Geisser adjustment was applied to correct for violations of sphericity. An analysis of variance (ANOVA) for repeated measurements with Greenhouse-Geisser adjustment was used for the treatment periods. Figures were generated using SigmaPlot (Systat Software inc.; San Jose, CA, USA). The local ethics committees of the Friedrich-Schiller University Jena and the Martin-Luther University Halle-Wittenberg independently approved the analysis.

## Results

### Cohort description

Data sets from 52 patients (47 female, 5 male) aged between 22 and 78 years (50.5 years, SD 12.63) were analyzed. Data on headache days in each individual month within the analyzed period were obtainable from 29 patients. We analyzed the admission forms patients had to fill out before the visit which contained headache days, intake of acute medication of the last three months during the regular screening follow-ups. As some patients (71.2%) were escalated after more than 3 months of treatment, post70 and pre140 intervals were defined as stated above. 5.8% (3/52) had 70 mg erenumab for 1 month, 11.5% (6/52) for 2 months and 37 patients more than 3 months.

The average number of headache days per month at baseline (pre70) was 15.67 days (± 6.37). 52% of the patients (*n* = 27) fulfilled the diagnostic criteria for chronic migraine. Data on acute medication at BL were missing for four patients (8%). Average acute medication was taken on 12.22 (± 5.39) days. Prior to the initiation of erenumab therapy, an average of 3.67 (± 1.06) unsuccessful drug treatments were established for prophylactic treatment. Twelve patients received ≥ 5 (23.1%), 17 at least 4 (32.7%), 11 at least 3 (21.6%), 7 at least 2 (13.5%), and one patient only had one previous preventive medication. Data from four patients concerning previous prophylactics was incomplete and thus not evaluated. There were contraindications for at least one preventive drug in 60% of all patients before erenumab initiation. 55.8% of the cohort (29/52) received at least one treatment attempt with onabotulinumtoxin A. Twenty-seven patients (56%) were diagnosed with MO at BL. Patients were treated for an average of 6.38 ± 4.31 months with 70 mg erenumab and 9.52 ± 7.47 consecutive months with erenumab 140 mg. Forty-two datasets could be retrieved for the 6 months period, 52 for the 3 months after erenumab 140 mg dose escalation.

28.8% of the datasets were missing monthly data and were not available, but average three months data could be evaluated in the screening visits.

### Comparison between study centers

In the Headache Center Jena 23 patients with a mean age of 49.21 years (± 10.58) and in Halle 29 patients with a mean age of 51.00 years (± 14.07) were analyzed. The data samples between Jena and Halle showed no significant difference between number of headache days at BL (16.17 MHD Halle vs. 15.03 MHD Jena pre 70 mg; *p* = .567), age (*p* = .746), usage of acute medication (*p* = .718) and gender (nearly 90% females in both centers). More details are given in Table 1.

**Table 1:** Demographic and headache related parameters of the study cohort

	Halle (*n* = 29)	Jena (*n* = 23)
age (years)	51.00 (SD = 13.82)	49.81 (SD = 10.35)
gender (female/male)	90/10%	87/13%
migraine type (CM/EM)	62/38%	35/65%
medication-overuse	58%	55%
previous migraine prophylaxis/attempts	3.69 (SD = 1.11)	3.63 (SD = 1.07)

Abbreviations: SD, standard deviation; CM, chronic migraine; EM, episodic migraine

### Number of Headache days

**Fig. 1:**
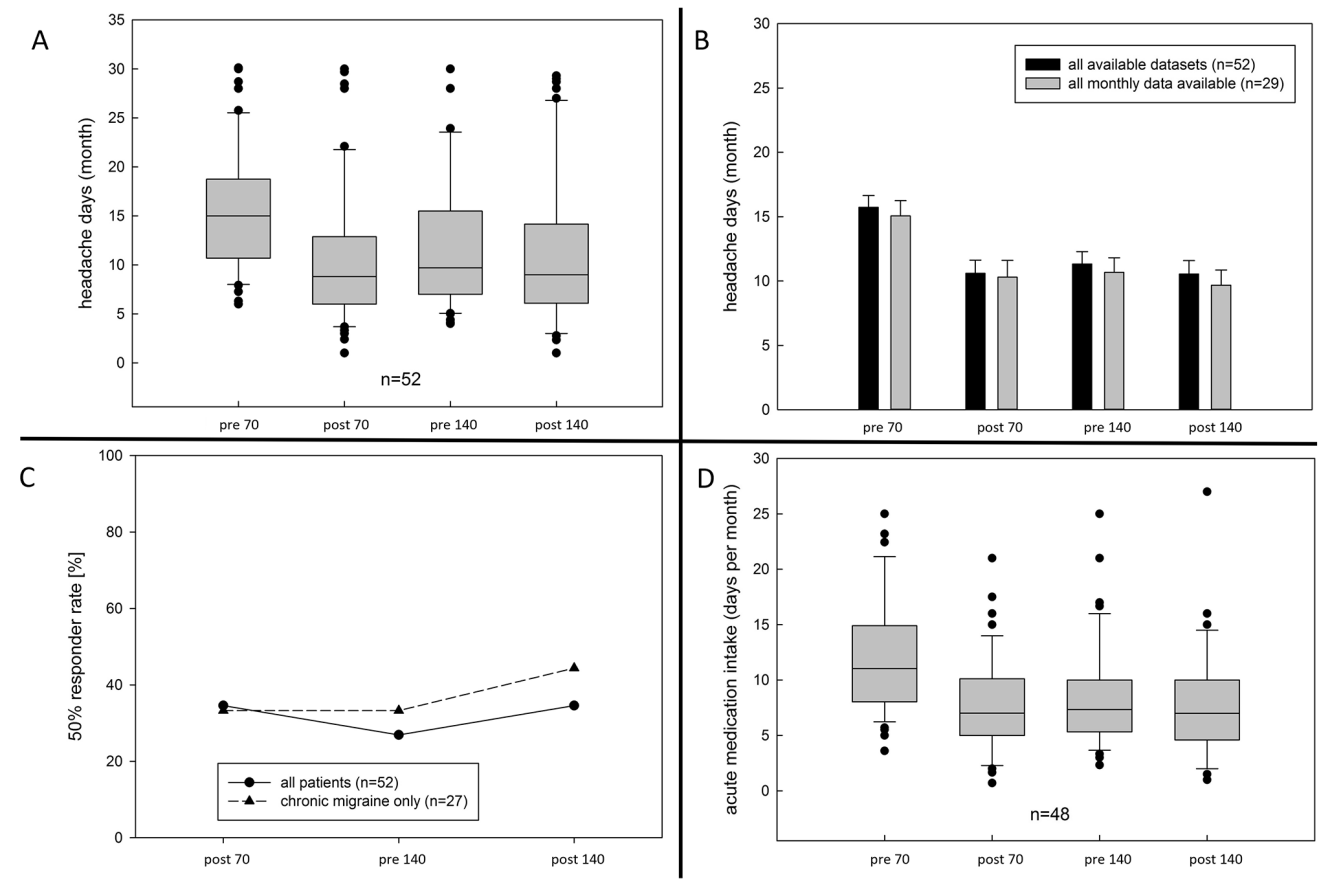
Clinical course after therapy escalation (70 mg to 140 mg). **A**: Course of headache days, **B**: Comparison regarding headache days of the full cohort and the cohort with complete datasets; **C**: 50% response; **D**: days with intake of acute medication. All time points represent averaged 3-month intervals. pre 70 = 3 months immediately before 70 mg erenumab, post 70 = 3 months directly following 70 mg erenumab treatment, pre 140 = 3 months immediately before dosage increase to 140 mg erenumab, post 140 = 3 months directly following 140 mg erenumab treatment

There was no difference in headache days comparing the patients with a complete dataset (data available for each month) and the whole cohort in which for some patients only the averaged data for the three months intervals were available (Fig. 1B).

An analysis of variance (ANOVA) for repeated measurements with Greenhouse-Geisser adjustment showed a significant effect by the time of measurement, *F*(2.01, 104.82) = 23.34, *p* < .001, partial η^2^ = 0.314.

**Fig. 2:**
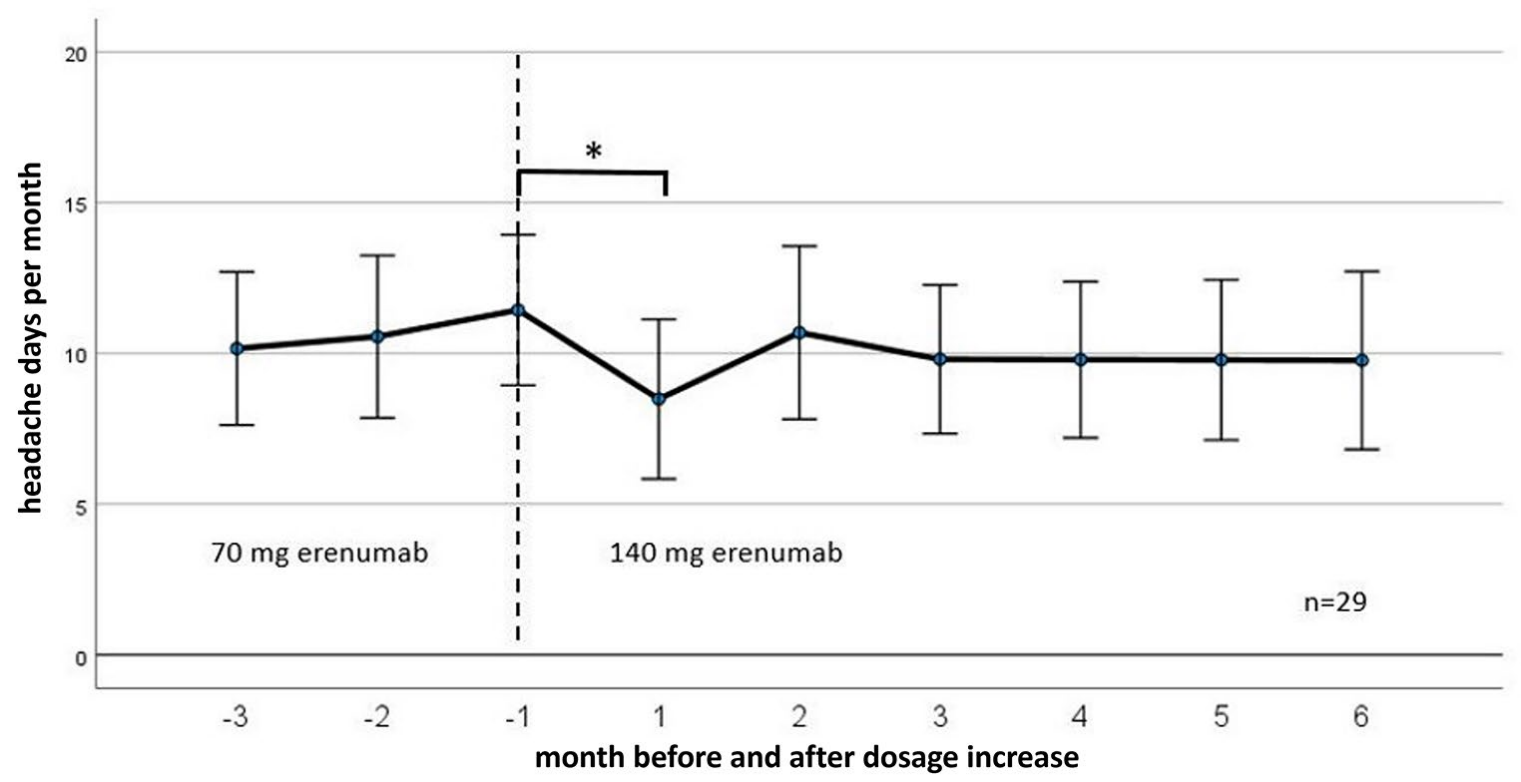
Illustration of monthly headache days with three months 70 mg to six months 140 mg erenumab treatment. Only patients with complete records were included in this figure. The numbers of the X-axis represent the months before (negative) and from (positive) the dose increase. In which − 1 corresponds to the retrospective analysis performed at the time of the dose increase

A strong effect size was found according to the classification by Cohen. Average headache days therefore significantly differed between time of measurements. Post-hoc-test with Bonferroni correction showed a reduction of 5.07 monthly headache days after treatment with 70 mg erenumab (*p* < .001, 95%-CI [3.35, 6.80]). The reduction of mean monthly headache days after treatment with 140 mg erenumab was − 4.5 headache days (*p* < .001, 95%-CI [2.09, 6.91]). No difference (− 0.47 headache days) was found between headache days pre 140 mg and post 140 mg (*p* = 1.00, 95%-CI[-0.82, 1.75]; Table 2; Figs. 1A and 2). ANOVA of the subgroup chronic migraine showed the same results (mean reduction of MHD − 6.41 days pre and post 70 vs. EM by the time of measurements).

**Table 2:** Summarized results regarding the primary and secondary endpoints

	pre 70 mg	post 70 mg	pre 140 mg	post 140 mg	*p*-Value
headache days (*n* = 52)	15.67 (± 6.40)	10.60 (± 7.11)	11.64 (± 6.92)	11.17 (± 7.91)	*p* < .001 (ANOVA)
headache days EM (*n* = 25)	10.57 (± 2.52)	6.94 (± 3.40)	8.84 (± 4.41)	9.12 (± 6.42)	*p* < .001 (ANOVA)
headache days CM (*n* = 27)	20.39 (± 5.13)	13.98 (± 7.99)	14.23 (± 7.85)	13.06 (± 8.77)	*p* < .001 (ANOVA)
acute medication intake (*n* = 47)	11.94 (± 5.17)	7.88 (± 4.35)	8.63 (± 4.67)	7.68 (± 4.85)	*p* < .001 (ANOVA)
acute medication intake EM (*n* = 25)	9.81 (± 2.64)	6.22 (± 3.28)	7.42 (± 3.56)	6.92 (± 3.87)	*p* < .001 (ANOVA)
acute medication intake CM (*n* = 22)	14.37 (± 6.24)	9.77 (± 4.70)	10.02 (± 5.44)	8.55 (± 5.73)	*p* < .001 (ANOVA)
medication overuse	56% (27/48)	27% (13/48)	31% (15/48)	29% (14/48)	*p* < .001 (Cochran-Q test)
50% response (*n* = 52)		35% (18/52)	25% (13/52)	35% (18/52)	*p* = .168 (Cochran-Q test)
50% response EM (*n* = 25)		36% (9/25)	16% (4/25)	25% (6/25)	*p* = .150 (Cochran-Q test)
50% response CM (*n* = 27)		33% (9/27)	33% (9/27)	44% (12/27)	*p* = .105 (Cochran-Q test)

Table 2: Time of measurements (pre 70 mg, post 70 mg, pre 140 mg, post 140 mg) on averaged 3 months data. Abbreviations: CM, chronic migraine; EM, episodic migraine; 50% response (reduction of 50% headache days 3 months before and after erenumab treatment)

Mean difference between pre 140 and post 140 mg in the chronic migraine subgroup was 1.16 ± 0.92 headache days (*p* = .394).

In the subgroup of episodic migraine there was merely a difference between pre and post 70 mg (*p* < .001). Before escalation to 140 mg was a rise of 1.9 mean headache days in EM (*p* < .092). There was no difference post 140 and pre 140 mg in EM (+ 0.28 MHD, *p* = 1.00; Table 2).

### Acute Medication Intake

Data regarding acute medication intake was missing in five patients of the overall cohort. An analysis of variance (ANOVA) showed a significant effect by the times of measurement with strong effect size, *F*(2.32, 106.47) = 26.04, *p* < .001, partial η^2^ = 0.361. Post-hoc test with Bonferroni correction showed a reduction of 4.07 intake days after 70 mg erenumab (*p* < .001, 95% -CI [2.62, 5.52]) compared to baseline (Fig. 1D). There was a reduction of 4.26 intake days post 140 mg erenumab compared to BL (*p* < .001, 95%-Cl[2.41;6.13]). Post 140 mg erenumab was not significant with a mean reduction of 0.95 intake days compared to pre 140 mg (*p* = .211, 95% -CI [-0.26, 2.17]).

The subgroups CM/EM did not differ from the total cohort in post hoc Bonferroni calculations.

### 50% response

50% response (compared to BL) was calculated for the above-mentioned periods. 35% of the total cohort (*n* = 52) showed a 50% reduction of MHD in the first three months after initiation of 70 mg erenumab (Fig. 1C). There was no difference between EM and CM (36% in EM vs. 33% in CM). Escalation from 70 mg to 140 mg erenumab did not significantly increase the rate of 50% response within the first three months, not just in our total study sample but also in the subgroups (pre 140 mg: 25%, post 140 mg: 35%, Cochran-Q test, *p* = .168, subgroups: episodic migraine 16% pre 140 mg 25% post, *p* = .150; chronic migraine 33% pre 140 mg, 44% post 140 mg *p* = .105, Cochran-Q Test).

### Medication overuse

56% of the patients (*n* = 27) used acute medications more than ten days per month over the three months baseline period. The rates of patients with MO differed between the analyzed time intervals: The rate of MO post 140 mg was twenty-nine, showing a significant reduction compared to BL (BL vs. post 140 mg, *p* < .001). We found no significant difference of MO rates between post 70 mg, pre 140 mg and post 140 mg time measurements (*p* < .001, Cochran-Q Test).

### Response of the non-responders

As treatment failure was not predefined, not all patients that were escalated to 140 mg had a response < 50%, but 75% of patients showed less than a 50% response after 70 mg erenumab initiation (39/52). This cohort was defined as non-responder and analyzed separately (Table 3). Headache days before and after the 140 mg erenumab escalation did not differ significantly (13.40 MHD pre 140 mg vs. 12.97 after 140 mg, *p* = .426, paired t-test). Further distinction between episodic and chronic migraine showed, comparably to the total cohort, also no significant difference (episodic: before 140 mg 9.55 MHD vs. 9.8 MHD after, *p* = .760; chronic: 17.89 MHD before vs. 16.67 MHD after 140 mg erenumab; *p* = .070, paired t-test). To note, in episodic migraineurs escalation to 140 mg erenumab did not decrease monthly headache days at all (+ 0.25). Regarding the secondary endpoint intake days of acute medication, no significant difference was found, neither in the total cohort nor in episodic and chronic migraineurs (9.58 intake days before and 8,73 intake days after; *p* = .190, paired t-test), and medication overuse was not reduced (41.67% vs. 36.11%; *p* = .527, Wilcoxon test). The 50% response of these non-responders was as little as 5.13% (2/39).

**Table 3:** Summarized results of the non-responders

	pre 140 mg	post 140 mg	*p*-Value
headache days (*n* = 39)	13.40 (± 7.13)	12.97(± 8.15)	*p* = .426 (paired t test)
headache days EM (*n* = 21)	9.55 (± 4.46)	9.8 (± 6.69)	*p* = .760 (paired t test)
headache days CM (*n* = 18)	17.89 (± 7.11)	16.67 (± 8.31)	*p* = .070 (paired t test)
acute medication intake (*n* = 36)	9.58 (± 4.81)	8.73 (± 4.81)	*p* = .190 (paired t test)
acute medication intake EM (*n* = 21)	8.05 (± 3.49)	7.42 (± 3.80)	*p* = .402 (paired t test)
acute medication intake CM (*n* = 15)	11.71 (± 5.66)	10.56 (± 5.58)	*p* = .156 (paired t test)
medication overuse (*n* = 36)	41.67% (15/36)	36.11% (13/36)	*p* = .527 (Wilcoxon test)

Table 3: Additional analysis of the patients with non-response defined as response to 70 mg erenumab regarding the headache days. This analysis directly compares the data of the three months before 140 mg erenumab escalation and the first 3 months under 140 mg erenumab therapy regarding headache days, days of intake of abortive medication and medication overuse. Abbreviations: CM, chronic migraine; EM, episodic migraine; SD, standard deviation

## Discussion

This study reconfirms effectiveness of erenumab 70 mg and 140 mg even in difficult-to-treat patients in a real-world treatment setting, and thus adding evidence to the already existing large, controlled clinical trials. Erenumab 70 mg and 140 mg reduce monthly headache days, as well as days of acute medication intake. This is in line with other real-world data around the globe underscoring the efficacy and safety of erenumab in patients with multiple treatment failures [11–15]. However, an increase of the dose from 70 mg to 140 mg erenumab apparently does not increase this efficacy in migraine patients any further.

These results are in line with the large clinical trials investigating 70 and 140 mg (Strive for EM and the Phase 2 CM trial), where both dosages were studied in parallel trial-arms and showed no significant differences regarding the primary outcome measures [3, 8]. We could not find superiority of 140 mg in our real-world data, as we could not find a positive effect of the dose escalation from 70 mg to 140 mg after 3 months of therapy concerning our primary outcome parameter (headache days) in the total cohort. Furthermore, the dose increase did not result in taking fewer acute medications. This reconfirms recent real-world data from Italy where erenumab showed to be effective in chronic migraineurs with > 4 previous treatment failures [12]. In this Italian study forty-three patients were escalated from 70 mg to 140 mg, but as dose escalation was performed at variable time intervals the authors were unable to assess the exact contribution of dose escalation to treatment effect.

Data focusing on dose increase are very sparse, but there is some evidence favoring the higher dose. Data from a 52-week open-label extension, phase-2 study support the benefit of the 140 mg erenumab in chronic migraineurs, as post-hoc analyses of efficacy parameters, including reductions in mean monthly migraine days, responder rates, and reduction in days of use of acute migraine-specific medications favored 140 mg. This does not strictly contradict our findings, as these were long-term data and dosage escalation was performed only in a subset of patients and not due to non-response, but per protocol. Furthermore, as only patients which completed the 52-week open label phase were included, these data may be biased by higher drop-out rates of non-responders [16]. A recent Canadian study showed that the treatment after dose increase was more likely to be continued than a deescalation from 140 mg to 70 mg or maintenance of 70 mg [17]. Furthermore, a recent systematic review investigating EM performed a pairwise meta- and Bayesian network analysis found a significant improvement in response rate and a reduction in monthly acute migraine-specific medication days following dose increase. This however, was in contrast to the lack of functional improvement after the higher dose was implemented. For safety, the analysis also found no significant difference between the two regimens [18]. Authors concluded that 140 mg may be a better choice for patients with episodic migraine with prior migraine treatment failure, but also stated that a direct comparison of dose increase after treatment failure of the lower dose would be highly warranted in further studies for final clarification.

In our study only very few findings are suggestive for some benefit of 140 mg. For example the 50% response rate following dosage escalation to 140 mg was numerically slightly higher in the CM subgroup (44% vs. 25% EM) and improvement in headache days of the CM non-responders trended towards significance (*p* = .070). However, in our study the 50% response of the 70 mg non-responders after escalation to 140 mg was as little as 5.14%, which numerically appears inferior to switching monoclonals antibodies according to a recent observational multicenter study [19]. Consequently, switching antibodies seems more promising than dose escalation of erenumab.

The main limitation of our study is the retrospective design to analyze the effect of a dose escalation. Further studies should engage in randomized, placebo controlled and maybe crossover designs in order to compare effects of 70 mg maintenance therapy versus 140 mg on the course of the disease.

One could argue, that the patients of both headache study centers were not equally balanced, but although rates of EM and CM differed between study centers (CM in Halle 62% vs. 35% in Jena), averaged headache days showed no significant differences (16.17 vs. 15.03 MHD), underlining the fact of an equally severe impacted and homogenic study group. Furthermore, at the two headache Centers different headache diaries were used, so distinct data including migraine days or headache intensity is missing. We did not screen for concomitant medication or the use of non-drug therapy. The time of the escalation to 140 mg varied between patients, depending on the erenumab 70 mg response, so more standardized data would be needed in order to distinguish between immediate responders and late onset responders. Further studies should also address migraine specific and non-specific acute medication intake, because risk of chronification seems to depend on dosage, medication class and migraine subtype [20, 21]. It would also be interesting how responses to specific acute medications evolve under erenumab treatment.

## Conclusion

Our study suggests that the dose increase from 70 mg to 140 mg erenumab in treatment refractory patients who did not respond 70 mg may not be successful. In this case switching monoclonal antibodies may be considered as an alternative. Further data are needed to evaluate effects of immediate and late onset erenumab escalation response in different subgroups.

## Declarations

PS, PB, TK and SN received lecture fees from Novartis. PS and SN were also provided with a research grant from Novartis.
